# A Case of False Negative NIPT for Down Syndrome-Lessons Learned

**DOI:** 10.1155/2014/823504

**Published:** 2014-02-04

**Authors:** Meagan Smith, Kimberly M. Lewis, Alexandrea Holmes, Jeannie Visootsak

**Affiliations:** Department of Human Genetics, Emory University, Atlanta, GA 30033, USA

## Abstract

Down syndrome or trisomy 21 is the most common cause of prenatal chromosome abnormalities with approximately 50% of all reported chromosome conditions. With the successful introduction of noninvasive prenatal testing (NIPT) for Down syndrome into routine prenatal care, it is important to understand the risks, benefits, and limitations in order to guide patients in making an informed decision. Herein, we describe the first published case report of a patient whose fetus tested “negative” for Trisomy 21 by NIPT but was diagnosed postnatally with trisomy 21. We present the importance of proper pretest and posttest genetic counseling to ensure prenatal patients are able to make informed decisions and are educated appropriately about NIPT.

## 1. Introduction

It has been a little over two years since noninvasive prenatal testing (NIPT) was introduced as part of prenatal care to screen high-risk patients for fetal aneuploidy in the United States. Since then, the clinical use of NIPT has become increasingly common as it provides women with a more sensitive noninvasive screening option with detection rates reported as high as 99% for Down syndrome (DS) or Trisomy 21 [1]. Other clinical noninvasive screening modalities for DS include the first trimester screen with a detection rate of 75–80% and the maternal serum screen which has a detection rate of 80% [1]. NIPT evaluates cell-free DNA (cfDNA) that circulates in maternal blood and can be done as early as 9-week gestation [2]. While NIPT has shown to be both highly sensitive and highly specific across numerous studies [1, 3–11], the only diagnostic tests offered prenatally for DS or other aneuploidies are amniocentesis and chorionic villus (CVS) sampling.

Several professional organizations, including the American College of Obstetrics and Gynecology (ACOG), the Society of Maternal Fetal Medicine (SMFM), and the National Society of Genetic Counselors (NSGC), have released position statements to help guide prenatal practices on the indications for use of NIPT [12, 13]. The overarching agreement among these organizations indicates that NIPT should only be offered to high-risk women as defined by (1) maternal age 35 years or older at delivery, (2) fetal ultrasound findings indicative of possible aneuploidy, (3) previous history of prior pregnancy with trisomy, (4) known familial robertsonian translocation, or a (5) previous positive prenatal screen [12, 13]. They also recommend NIPT testing only be given in context with pretest and posttest genetic counseling [12, 13]. Further, the American College of Medical Genetics (ACMG) recently recommended that the term NIPT be replaced by noninvasive prenatal screening (NIPS) as NIPT is not a diagnostic test and positive screening results should be confirmed by an invasive diagnostic procedure [14].

Herein, we describe the first published case report of a patient whose fetus tested “negative” for Trisomy 21 by NIPT but was diagnosed postnatally with Trisomy 21. We present the importance of proper pretest and posttest genetic counseling to ensure prenatal patients are able to make informed decisions and are educated appropriately about the benefits and limitations of NIPT.

## 2. Clinical Report

The patient is a 3-month old female who presented to our clinic with her 33-year old mother and 32-year old father. The patient's mother reported that this was her third child and that her two previous pregnancies were uneventful and resulted in the birth of two healthy children who are developing appropriately for their ages. The parents report that the patient was born at 37-week 4-day gestation by elective cesarean section with a birth weight of 7 pound 3 ounces (3.26 kg). Down syndrome (DS) was suspected based on physical features seen at birth (hypotonia, flattened nasal bridge, and upslanting palpebral fissures), and a chromosome analysis confirmed the diagnosis of DS, 47,XX,+21 (6 cells analyzed and 3 cells karyotyped, mosaicism ruled out). Complications seen in the neonatal period included respiratory distress with noted transient tachypnea that required supplemental oxygen and a complete atrioventricular canal defect (CAVCD) detected on the newborn echocardiogram.

The patient's mother reported that the prenatal history for this pregnancy was relatively uncomplicated, with normal ultrasound finding throughout the pregnancy until 20-week gestation, when a CAVCD was detected. The patient's mother was offered an amniocentesis but declined the invasive diagnostic testing in favor of the noninvasive option, Verifi © NIPT. When the results were reported as “no aneuploidy detected,” the patient's parents concluded that these results were reassuring news that the fetus was negative for the trisomies that are currently screened for by this test (trisomy 21, 18, and 13) [15].

At the time of the visit the family was trying to come to terms with their child's diagnosis of DS, while still trying to understand the meaning of the discordant negative NIPT result.

## 3. Discussion

It is a common misconception among patients that NIPT is diagnostic in value. These beliefs are often based on the misconceptions formed by seeing advertised testing sensitivity and specificity reported as >99-100% (Table 1) [1, 6, 16–20]. While it is true that the sensitivity and specificity of the new NIPT are higher than those reported with traditional first trimester screening or multiple marker screening methods, it is imperative for medical professionals and their patients to understand that NIPT is still a screening tool and cannot replace the high level of accuracy seen by diagnostic testing. The sensitivity and specificity reported by many of companies offering NIPT are based on validation studies of only a few hundred to thousand individuals and have not factored in data obtained in the clinical setting. For this reason, all patients should be counseled prior to testing on the various possible test results as the risk of false positive or false negative results can occur.

Much like traditional screening methods, NIPT test results are categorized into low risk of aneuploidy and high risk of aneuploidy, as well as no-call (undeterminable). Individuals who are determined to be in the low-risk category would not require any further diagnostic evaluation, unless warranted by other clinical findings. Individuals who are determined to be in the high-risk category should be offered confirmatory diagnostic testing (i.e., chorionic villus sampling or amniocentesis). The no-call category is used to categorize samples that did not generate a result and would require a repeat test sample to be drawn. False-positive results can occur in the presence of placental mosaicism, vanishing twin syndrome, or an unidentified maternal condition, such as mosaicism or cancer. False negative results can occur when an insufficient amount of fetal cfDNA is present in the sample, resulting in masking on the fetal phenotype by the maternal cfDNA.

When considering NIPT it is not only important for the patient to receive appropriate pretest counseling on the benefits and limitations of this new technology, but for medical professionals to ensure that testing is offered only when appropriate. Advertised indications for testing released by three out of the four companies (Verinata Health (Redwood, CA, USA), Natera (San Carlo, CA, USA), and Sequenom Inc. (San Diego, CA, USA)) currently offering NIPT, are in agreement with published position statements stating that testing should only be offered to patients with a singleton pregnancy who are deemed high risk (as previously defined) [12, 13]. The use of NIPT outside of these designated settings has not been validated and therefore is not recommended because the accuracy of the results is unknown at this time.

As the medical community continues to embrace new technologies and incorporate them into daily clinical practice, it is imperative to ensure that the appropriate level of education is occurring for the provider ordering the test and the patient being offered the test. When knowledgeable medical professionals properly discuss the utility of NIPT and provide patients with anticipatory guidance regarding the possible outcomes, they enable the patient to make a more informed decision regarding the role of NIPT in their pregnancy.

## Figures and Tables

**Table 1:** Commercially advertised NIPT statistics [1, 6, 16–20].

	Sequenom MaterniT21 PLUS	Verinata Verifi	Ariosa Harmony	Natera Panorama
Sensitivity	98.6%–99% (209/212)	>99% (90/90)	100% (81/81)	>99% (25/25)
Specificity	99.8% (1468/1471)	99.8% (409/410)	99.97% (2887/2888)	>99% (242/242)
False positive	0.2% (3/1471)	0.2% (1/410)	0.03% (1/2888)	0
No call rate	3.4%	5.8%	4.7–5.7%	5.4%
